# Interleukin-6 Is a Biomarker for the Development of Fatal Severe Acute Respiratory Syndrome Coronavirus 2 Pneumonia

**DOI:** 10.3389/fimmu.2021.613422

**Published:** 2021-02-18

**Authors:** André Santa Cruz, Ana Mendes-Frias, Ana Isabel Oliveira, Luís Dias, Ana Rita Matos, Alexandre Carvalho, Carlos Capela, Jorge Pedrosa, António Gil Castro, Ricardo Silvestre

**Affiliations:** ^1^Life and Health Sciences Research Institute (ICVS), School of Medicine, University of Minho, Braga, Portugal; ^2^ICVS/3B's—PT Government Associate Laboratory, Guimarães, Portugal; ^3^Department of Internal Medicine, Hospital of Braga, Braga, Portugal

**Keywords:** COVID-19, IL-6, SARS-CoV-2, fatal pneumonia, biomarker

## Abstract

Hyper-inflammatory responses induced by severe acute respiratory syndrome coronavirus 2 (SARS-CoV-2) are a major cause of disease severity and death. Predictive prognosis biomarkers to guide therapeutics are critically lacking. Several studies have indicated a “cytokine storm” with the release of interleukin-1 (IL-1), IL-6, and IL-8, along with tumor necrosis factor alpha (TNFα) and other inflammatory mediators. Here, we proposed to assess the relationship between IL-6 and outcomes of patients with coronavirus disease 2019 (COVID-19). Our cohort consisted of 46 adult patients with PCR-proven SARS-CoV-2 infection admitted in a COVID-19 ward of the Hospital de Braga (HB) from April 7 to May 7, 2020, whose IL-6 levels were followed over time. We found that IL-6 levels were significantly different between the disease stages. Also, we found a significant negative correlation between IL-6 levels during stages IIb and III, peripheral oxygen saturation (SpO_2_), and partial pressure of oxygen in arterial blood (PaO_2_), showing that IL-6 correlates with respiratory failure. Compared to the inflammatory markers available in the clinic routine, we found a positive correlation between IL-6 and C-reactive protein (CRP). However, when we assessed the predictive value of these two markers, IL-6 behaves as a better predictor of disease progression. In a binary logistic regression, IL-6 level was the most significant predictor of the non-survivors group, when compared to age and CRP. Herein, we present IL-6 as a relevant tool for prognostic evaluation, mainly as a predictor of outcome.

## Introduction

Coronaviruses are a family of single strain RNA viruses that infect several hosts, including humans, mainly causing respiratory infections (1). Severe acute respiratory syndrome coronavirus 2 (SARS-CoV-2), a novel betacoronavirus, emerged at the end of 2019 in China and has already infected almost 90 million people worldwide, causing more than 1.9 million deaths and becoming a worldwide pandemic (coronavirus disease 2019, COVID-19) (2, 3). Although most cases present only mild symptoms, 20% of the patients develop severe pathology with acute bilateral pneumonia that may evolve to acute respiratory distress syndrome and multi-organ failure. The risk of severe disease and death increases with age and the presence of comorbidities (4).

Infection with SARS-CoV-2 comprehends two overlapping phases: the first, characterized by a high replicative activity of the virus, is then followed by a counteractive host immune response (5). This infection has been divided into three clinical stages, regarding the severity and prognosis (6, 7). Stage I is defined by mild unspecified symptoms, such as myalgia, dry cough, headache, and subfebrile temperature, without any laboratory and radiological abnormalities. Stage II is characterized by cough, high fever, dyspnea, abnormal thoracic imaging, lymphopenia, and increased levels of inflammatory markers. It is further divided into two groups, according to the presence (IIb) or absence (IIa) of hypoxemia. Finally, stage III displays clinical manifestations of a severe systemic inflammatory syndrome, culminating in severe respiratory failure with an unfavorable prognosis. During this last stage of the disease, values of several inflammatory markers are extremely high and macrophage activation syndrome may occur.

Several treatments for COVID-19 have been tested, which can be divided into three main categories: drugs with direct antiviral effect, drugs with immunomodulatory effect, and neutralizing antibodies from convalescent plasma (8). So far, among the first group, remdesivir has been considered the most prominent drug due to the evidence of faster clinical improvement and mortality reduction in the subset of hospitalized patients receiving oxygen (9, 10). However, these data are conflicting with other studies, and doubts remain about treatment efficacy and profile of patients that may benefit the most from this therapeutic (11, 12).

Considering the challenge of controlling virus transmission, and the lack of an unquestionably effective antiviral treatment, a therapeutic strategy of immunomodulation has been advocated (13). This strategy is particularly relevant given the excessive production of proinflammatory cytokines recognized as crucial in the pathophysiologic process of severe COVID-19 (14). In these cases, the loss of negative feedback in the immune response causes excessive production of inflammatory cytokines, leading to deleterious effects and poor prognosis (15). A large group of cytokines has been recognized as significantly increased in severe COVID-19 patients: interleukin-1β (IL-1β), IL-1RA, IL-2, IL-6, IL-7, IL-8 (CXCL8), IL-9, IL-10, IL-17, IL-18, tumor necrosis factor (TNF-α), interferon-gamma (IFN-gamma), granulocyte colony-stimulating factor (G-CSF), granulocyte-macrophage colony-stimulating factor (GM-CSF), macrophage inflammatory protein 1 (MIP-1alpha/CCL3), monocyte chemoattractant protein-1 (MCP-1/CCL2), interferon gamma-induced protein 10 (IP-10/CXCL10), and fibroblast growth factor (FGF) (16–18). Most importantly, some of them (IL-6, IL-8, and TNF-α) are regarded as independent markers of the severe disease (19). A deeper knowledge of the SARS-CoV-2-induced cytokine storm, including its triggering mechanisms, molecular components, and kinetics, is necessary for a better understanding of the pathological process in COVID-19 and therefore for the identification of the most adequate therapeutic targets and timing of drugs administration. So far, several studies have been published on the potential effects of specific (anti-IL-6, anti-IL-1, anti-GM-CSF, and anti-TNF-α) and non-specific therapies (corticosteroids) (13, 20). Among the immunomodulatory therapies for COVID-19, corticosteroids have been the most widely used, particularly dexamethasone, after growing evidence of their benefit in reducing mortality in hospitalized patients receiving oxygen and especially in patients supported with mechanical ventilation (20, 21). Nevertheless, the most adequate dosage for each patient, precise timing of administration, and duration of treatment remain to be elucidated. Also, a more selective drug would be desirable, especially considering the already existing immune dysfunction.

Of all the upregulated cytokines that may represent selective therapeutic targets, IL-6 has been regarded as particularly important in the COVID-19 pathogenesis and may be antagonized by existing drugs. IL-6 is an inflammatory interleukin mainly produced by macrophages and T lymphocytes in response to pathogens and is pivotal to controlling several viral infections (22–24). While homeostatic values of IL-6 contribute to the resolution of infections and tissue lesions, its exacerbated production contributes decisively to cytokine storms (22–24). In COVID-19, IL-6 has been positively correlated with disease stages and radiologic changes (17, 25–27). Furthermore, the potential prognostic value of IL-6 has been explored regarding the need for mechanical ventilation, mortality, or both, when considered alone or in combination with other variables (28–32). Yet, most studies quantify IL-6 only at patient admission, a strategy that may not be appropriate to accurately predict the outcome or to guide treatment due to the dynamic inflammatory process occurring during infection with SARS-CoV-2. Of all the available drugs that specifically inhibit IL-6 pathway, only tocilizumab (an IL-6 receptor antagonist) has, so far, a reasonable body of evidence in COVID-19. A recently published meta-analysis on the efficacy of tocilizumab in those patients found that cumulative evidence from randomized controlled trials (RCTs) suggests a risk reduction of mechanical ventilation but no effect on mortality, while cumulative evidence from cohort studies suggests an association between tocilizumab and lower mortality (33). However, only 3 of the 19 cohort studies and none of the 5 selected RCTs, used elevated IL-6 level as an inclusion criterion. This fact suggests that tocilizumab and other IL-6R antagonists may be further exploited.

In our work, we performed a characterization of the serum IL-6 levels throughout the entire infectious process with SARS-CoV-2. The IL-6 levels increase according to the disease stage and correlate with respiratory failure. After a kinetic analysis, we showed that the levels of IL-6 may be just temporarily raised, which may have major therapeutic implications. Moreover, the kinetic quantification of IL-6 levels allowed early discrimination between survivors and non-survivors. Overall, we suggest that a kinetic IL-6 quantification is crucial to predict the outcome of patients infected with SARS-CoV-2 and may be very useful to guide treatment.

## Methods

### Patients and Study Design

This is a single-center prospective cohort study, performed at Hospital de Braga (HB), a tertiary Portuguese Hospital. All adult patients with PCR-proven SARS-CoV-2 infection admitted in a COVID-19 ward from 7th April to 7th May 2020 were treated and monitored according to the HB protocol, which was approved by the Clinical Board and Ethics Committee (reference 69_2020). Among other recommendations, this protocol provides guidance on laboratory tests and includes the monitoring of IL-6 serum levels to all patients. Thirty out of the 46 enrolled patients were subjected to a kinetic serum IL-6 quantification at admission and on each 72 h, throughout hospitalization. This study ended when the last patient of this group was discharged. Patients that did not completely follow the protocol, who had evidence of any simultaneous bacterial infection, or patients treated with tocilizumab were excluded from this study.

### Interleukin-6 Quantification

Whole blood was collected in tubes containing separation gel (VACUETTE) and transported to the Life and Health Sciences Research Institute (ICVS) laboratories to further analysis. After centrifugation, the serum was collected and stored at −80°C. IL-6 was quantified using an ELISA kit (reference 430504, BioLegend, CA, USA), according to the manufacturer's instructions. All other laboratory tests, included in the established protocol, were performed in the HB laboratories, following the standard procedures.

### Data Collection

For each patient, data were collected from the medical records and inserted into our study database. Variables comprised of demographics, major comorbidities, disease symptoms, dates of onset, diagnosis, hospital admission, discharge, or death. At baseline and during hospitalization, daily information on the disease stage, existence of fever, peripheral oxygen saturation (SpO_2_), partial pressure of oxygen in arterial blood (PaO_2_), radiologic severity index, need for invasive or non-invasive ventilatory support, treatment used, diagnosis of a pulmonary embolism if present, C-reactive protein (CRP) quantification, and clinical impression of patients' evolution were collected.

### Statistical Analysis

Statistical analyses were performed using GraphPad Prism version 6 software. Regarding the small sample size and the non-normality observed in our variables, the Kruskal–Wallis test was used to identify statistical differences. For variables that reached global significance, pairwise comparisons were performed by the Mann–Whitney U-test. Correlations were calculated using Spearman's correlation: Spearman coefficient and *p-*value were reported. For categorical variables, the chi-square test was performed to assess the dependence between variables: Cramer's V and *p*-value were reported for each comparison. Binary logistic and linear regressions were performed using IBM SPSS statistics 26. Statistically significant values are as follows: ^*^p < 0.05; ^**^p < 0.01; ^***^p < 0.001.

## Results

### Demographic and Clinical Characterization of the Cohort

Of the 63 patients admitted in the COVID-19 ward during the period of the study, 17 were excluded: 10 due to evidence of simultaneous bacterial infection, 5 due to treatment with tocilizumab, and 2 due to insufficient compliance with the protocol. Our final cohort included the remaining 46 patients whose demographic and clinical characteristics are detailed in Table 1. Most patients were hospitalized between the 4th and 10th day of symptoms, with cough being the most prevalent complaint. Two-thirds of the patients were admitted due to bilateral pneumonia with hypoxemia (stage IIb or III). At admission, 41 patients (89%) reported symptoms related to COVID-19. Half of the patients who were admitted in stage IIb eventually progressed to stage III, with most of them requiring ventilatory support. Out of all the patients, five deaths were observed. All the other patients were discharged or transferred to the general ward after meeting cure criteria (complete resolution of the symptomatology and two negative PCR-SARS-CoV-2 results within 24–48 h).

**Table 1:** Demographic and clinical characterization of the cohort.

**Parameter**	***N* or mean (% or range)**
**Gender**, ***n (%)***	
Female	22 (48)
Male	24 (52)
**Age, years (range)**	69 (41–96)
**Underlying diseases**, ***n (%)***	
Autoimmune Disease	3 (6)
Immunosuppressed	2 (4)
Cancer history	7 (15)
Chemotherapy	1 (2)
Hypertension	31 (67)
Diabetes	18 (39)
COPD	3 (7)
Asthma	2 (4)
Other respiratory disease	4 (9)
Chronic Liver Disease (Child B)	1 (2)
Chronic Kidney Disease	7 (15)
**Symptoms**	
Days of symptoms before admission	7.41 (0–21)
Cough before admission	28 (61)
Dyspnea before admission	22 (48)
Fever before admission	27 (59)
**Reasons for admission**	
Hypoxemia	30 (65)
Relevant Constitutional Symptoms	4 (9)
Vomiting and Diarrhea with electrolyte imbalance	2 (4)
Other medical conditions	7 (15)
Surgical conditions	3 (7)
**Stage of disease at admission**	
Stage I	5 (11)
Stage IIa	11 (23)
Stage IIb	27 (59)
Stage III	3 (7)
**Treatment**	
Patients supported with non-invasive ventilation at any point	12 (26)
Patients supported with invasive ventilation at any point	6 (13)
No medication to the infection	10 (22)
Hydroxychloroquine (monotherapy)	9 (20)
Hydroxychloroquine + azithromycin	19 (41)
Hydroxychloroquine + Lopinavir + Ritonavir	3 (7)
Azithromycin (monotherapy)	1 (2)
Any of the above + corticosteroids	6 (13)
Corticosteroids (monotherapy)	4 (9)
**Outcome**	
Deaths	5 (11)
Cured	41 (89)

*COPD, Chronic Obstructive Pulmonary Disease*.

### Interleukin-6 as a Predictive Marker of Disease Progression and Respiratory Failure

**Figure 1:**
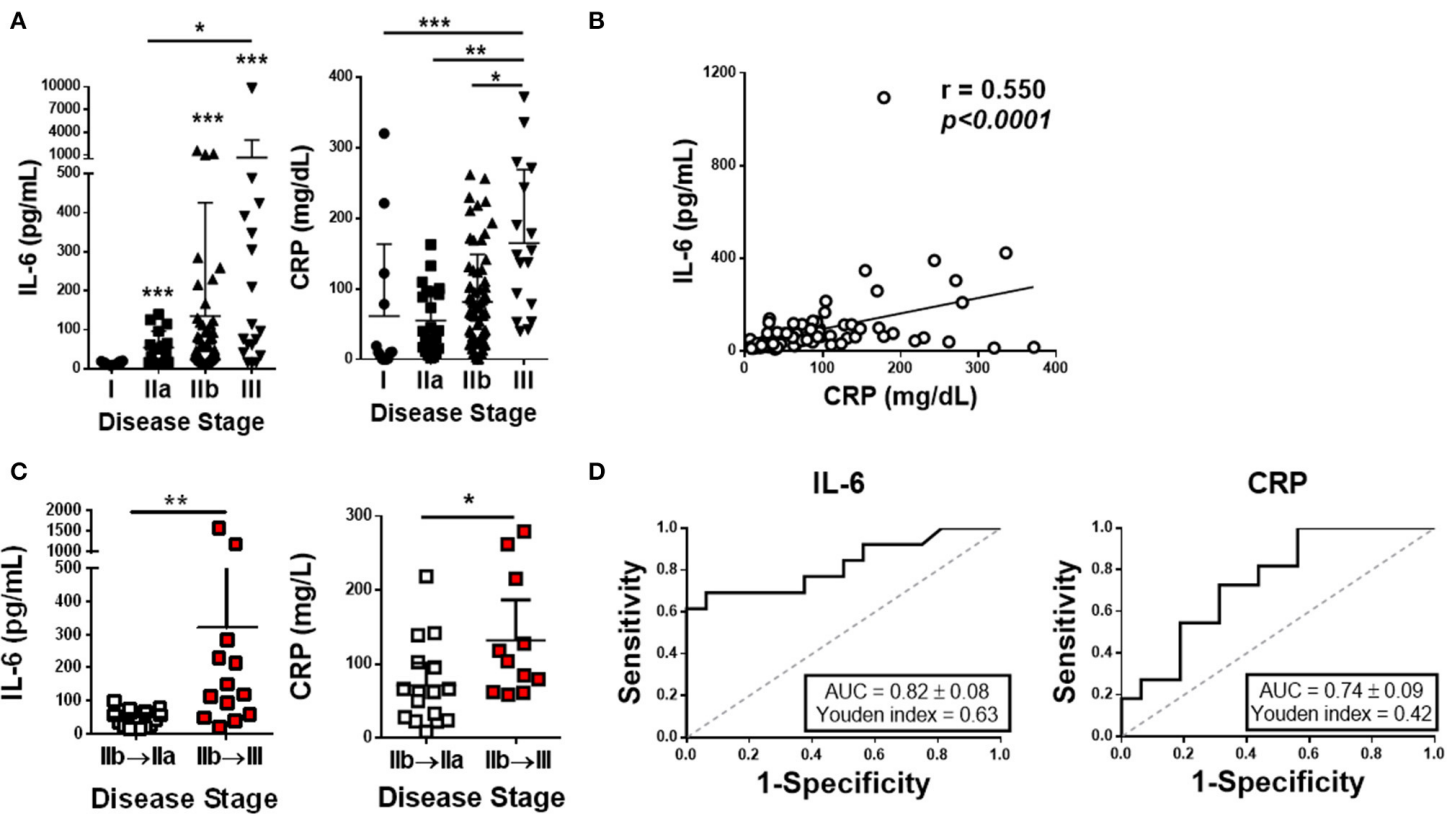
Plasma IL-6 and CRP profile in COVID-19 patients. **(A)** The levels of IL-6 and CRP were quantified on the plasma of COVID-19 patients segregated by disease stages. **(B)** Correlation between the plasma IL-6 and CRP levels in all patients. **(C)** IL-6 and CRP plasma concentration in patients at stage IIb that move to stage IIa or III. **(D)** ROC curves of IL-6 and CRP. In **(A,B)**, we have included multiple data from each patient (*n* = 46). Data are shown as mean ± SD ^*^p < 0.05, ^**^p < 0.01, ^***^p < 0.001.

Serum samples from subjects enrolled in our study were used for IL-6 quantification. After dividing IL-6 values according to disease stages (I, IIa, IIb, and III), statistical differences were found, with (*p* < 0.0001) IL-6 levels increasing along with the disease stage (Figure 1A). IL-6 values from patients in stage I are significantly lower than the values observed in the other stages (*p* = 0.0234, *p* = 0.0002, and *p* < 0.0001, compared to stages IIa, IIb, and III, respectively). The levels of CRP throughout the disease stages were also evaluated (Figure 1A). CRP shows a different pattern, as CRP levels were found significantly increased in stage III when compared to the other stages (*p* = 0.0002, *p* = 0.0015, and *p* = 0.0142 to I, IIa, and IIb, respectively). To evaluate the relationship between these two parameters, a Spearman's correlation was performed, and a positive correlation was found between IL-6 and CRP (*r* = 0.550, *p* < 0.0001; Figure 1B). Regarding the overall correlation of IL-6 levels with the increased severity of the disease, we hypothesized that IL-6 values could predict the disease progression of patients in the crucial IIb stage. In fact, we found that IL-6 levels were significantly higher in patients in stage IIb who will evolve to stage III, as compared to patients in stage IIb who will recover from their respiratory failure and enter stage IIa (*p* = 0.0022). These differences were also observed with CRP values (*p* = 0.0343; Figure 1C). These results were also corroborated by a receiver operating characteristic (ROC) curve, more robust for IL-6 when compared to CRP [area under the curve (AUC) = 0.82 ± 0.08 and Youden's index = 0.63 for IL-6 and AUC = 0.74 ± 0.09 and Youden's index = 0.42 for CRP; Figure 1D]. Of note, a linear regression was performed to assess the influence of any comorbidity in IL-6 levels, ruling out that hypothesis.

**Figure 2:**
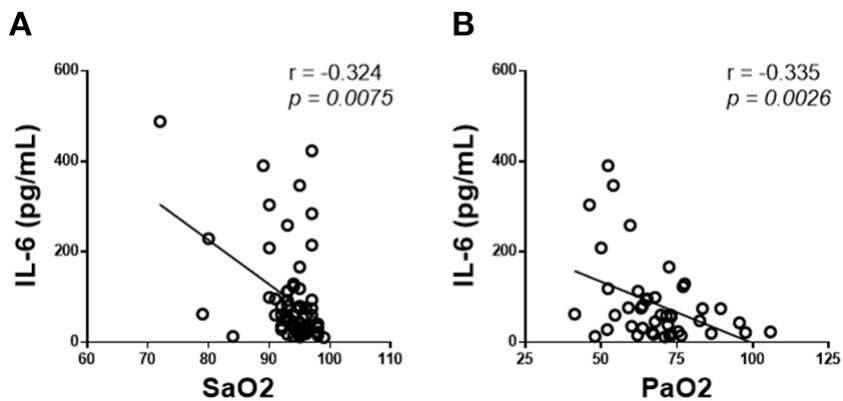
IL-6 correlation with respiratory parameters in COVID-19 patients. Correlation of plasma IL-6 levels with **(A)** oxygen saturation (SpO_2_) and **(B)** oxygen partial pressure (PaO_2_). We have included multiple data from each patient (*n* = 46).

In stages characterized by hypoxemia (IIb and III), IL-6 levels did correlate with patient's respiratory failure severity, as we observed a significant negative correlation with SpO_2_ (*r* = −0.324, *p* = 0.0075) and a significant negative correlation with PaO_2_ (*r* = −0.335, *p* = 0.0026) (Figures 2A,B).

### Interleukin-6 as a Prognostic Marker for Survival

**Figure 3:**
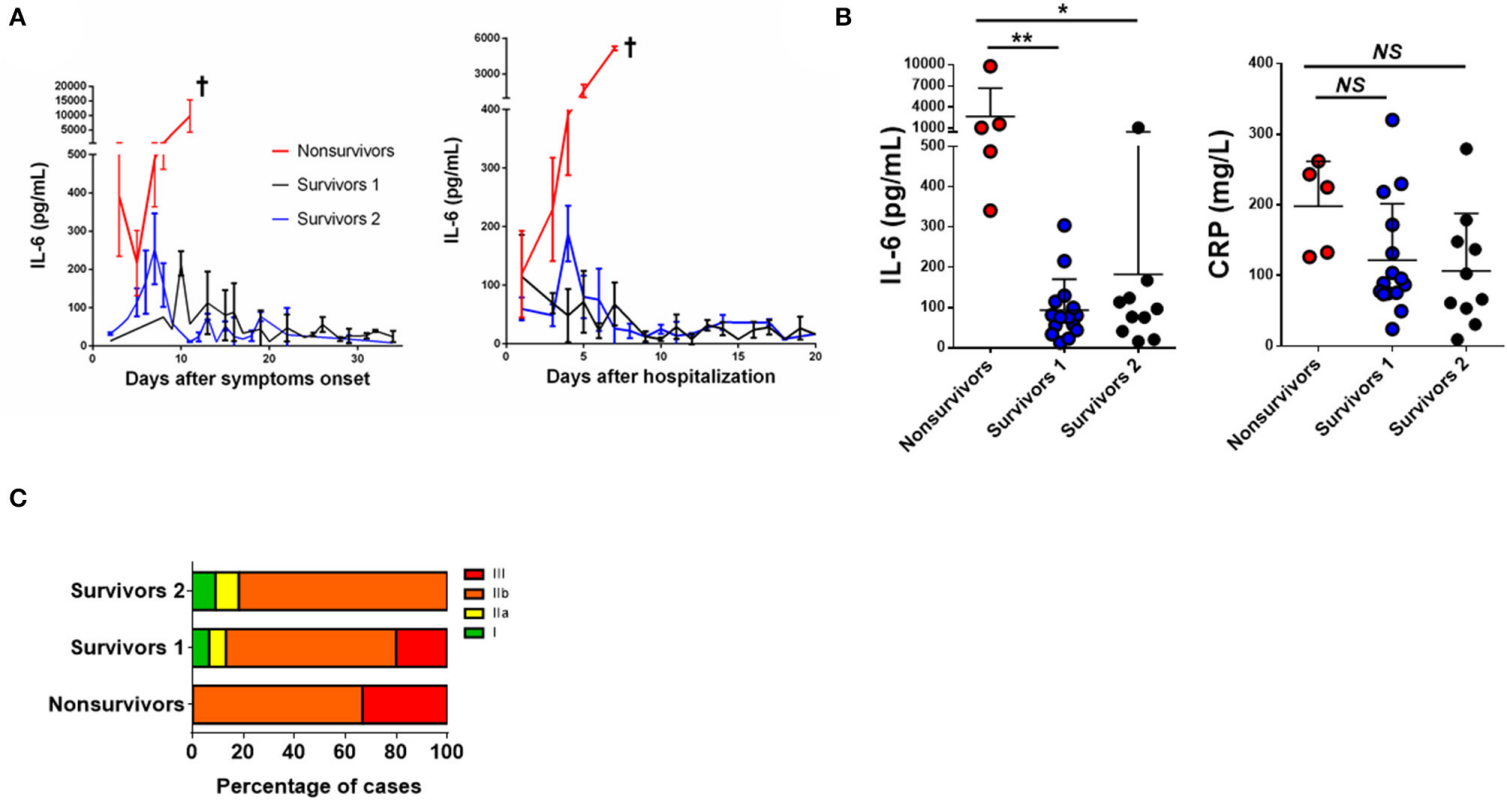
IL-6 as a mortality predictor in COVID-19 Patients. **(A)** Kinetic analysis of plasma IL-6 concentrations in COVID-19 patients assembled by days after the onset of symptomatology and after hospitalization. Data are represented as the median of plasma IL-6 levels in the non-survivors' group (red *n* = 5) and two distinguished groups of survivors (blue *n* = 15 and black *n* = 10). **(B)** Plasma levels of IL-6 and CRP. Data depict the number of patients as shown in Table 2, where each dot represents the highest IL-6 level for each patient during hospitalization. **(C)** Distribution among the disease staging at patient admission assigned to the three groups of non-survivors and survivors. Data are shown as mean ± SD ^*^p < 0.05, ^**^p < 0.01. †patient's death.

We depicted the IL-6 kinetics throughout the infection based on the onset of symptoms and the admission day. Patients were then grouped according to the shape of their IL-6 curve, as represented in Figure 3A. Matching profile and outcome, all patients in profile 1 (red line) died in the first week of hospitalization (non-survivors). All patients in the other profiles survived. In survivors' group 1 (black line), a peak of IL-6 is observed around day 10 after the onset of symptoms, but after admission, IL-6 levels decreased gradually as patients recovered. In survivors' group 2 (blue line), a peak of IL-6 is observed approximately at day 7 after the onset of symptoms and is also detected around day 4 of hospitalization, followed by decreasing values of IL-6 as patients recovered. It shall be noted, however, that patients in the survivors' group 1 were admitted 3 days later (median difference) than patients in the survivors' group 2, counting from disease onset. In both groups, all individuals displayed a peak of IL-6, which was limited in time. Importantly, after the 10th day of hospitalization, all these patients showed an IL-6 value close to normal.

Interleukin-6 and CRP levels were evaluated between the three profiles (Figure 3B). IL-6 levels of non-survivors were significantly higher when compared to the survivors' groups (*p* < 0.0033 and *p* = 0.0131; for survivors' group 1 and survivors' group 2, respectively), while no significant differences in CRP values were observed among the three profiles. Between the three profiles, there were different distributions of patients between the disease stages: in non-survivors, there were only patients in IIb and III stages, while in both the survivor's groups, patients in the stages I and IIa were also found (Figure 3C).

To identify if these three profiles could be explained by non-infection-related parameters, such as gender, underlying diseases, or treatment, a chi-square dependence test was performed between the profiles and these parameters (Table 2). This is a 3 × 2 chi-square test, thus the *p*-value was adjusted, being considered dependence only in comparations where *p* < 0.0042 and with adjusted residual (radj) higher than 2.635. As such, only the treatment with hydroxychloroquine + azithromycin influences the profile (*p* = 0.0003). However, this dependence is observed only between this treatment and survivors' group 1 and survivors' group 2 (r_adj_=|3.3| and r_adj_=|2.9|, respectively), and it is not observed with the non-survivors' group (r_adj_=|0.8|). Age was also evaluated between profiles: the non-survivors' group has a median age of 86 ± 21 years, the survivors' group 1 has a median age of 64 ± 32 years, and the survivors' group 2 has a median age of 73 ± 21 years. There were no significant differences between these three profiles (*p* = 0.056).

**Table 2:** Demographic and clinical characterization among different profiles.

**Parameter**	**Non-survivors (*n =* 5)**	**Survivors 1 (*n =* 15)**	**Survivors 2 (*n =* 10)**	**Qui-Square**	
				**Cramer's V**	***p-*value**
**Gender**, ***n (%)***					
Female	1 (20)	4 (27)	7 (70)	0.392	0.099
Male	4 (80)	11 (73)	3 (30)		
**Underlying diseases**, ***n (%)***					
Autoimmune Disease	–	–	1 (9)	–	–
Active Neoplasia	1 (20)	3 (20)	1 (9)	0.126	0.787
Asthma	–	1 (7)	–	–	–
Chronic Kidney Disease	–	3 (20)	2 (18)	0.200	0.549
COPD	1 (20)	1 (7)	2 (18)	0.248	0.396
Diabetes Mellitus	2 (40)	4 (27)	3 (27)	0.200	0.549
Hypertension	3 (60)	9 (60)	7 (70)	0.098	0.866
**Treatment**, ***n (%)***					
No Treatment	0	3 (20)	2 (20)	0.200	0.549
Corticosteroids	3 (60)	2 (13)	2 (20)	0.394	0.097
Hydroxychloroquine	1 (20)	0	4 (40)	0.482	0.031
Hydroxychloroquine+Azithromycin	2 (40)	13 (87)	2 (13)	0.629	0.003

*COPD, Chronic Obstructive Pulmonary Disease*.

Finally, we performed a binary logistic regression to predict the non-survivors' profile. Our model included the variables age, IL-6, and CRP, with a chi-square =24.856, Nagelkerke's R square = 0.752, and *p* < 0.0001. As presented in Table 3, IL-6 is the most significant variable to predict the non-survivors' profile (*p* = 0.0430).

**Table 3:** Logistic binary regression for prediction of profile 1.

**Variables**	**B**	**S.E**.	**Wald**	**df**	**sig**	**Exp(B)**
Age	0.057	0.055	1.067	1	0.302	1.058
CRP	−0.051	0.030	2.865	1	0.091	0.950
IL-6	0.052	0.026	4.091	1	0.043	1.053
Constant	−9.579	5.035	3.620	1	3.620	0.000

## Discussion

There is a high variability across studies in terms of characteristics and outcomes of patients with COVID-19, as these results are influenced by the countries' demographics, clinical settings, and health-systems' available resources. Regarding the studies already published, the best match for ours is a large Spanish cohort, with a similar setting (34). Compared to that study, our cohort was older (median age 8 years higher) and presented more comorbidities; distribution according to sex was very similar (52 vs. 48% males), as was the time from disease onset to admission (7 vs. 6 days). Although the number of patients in our group with respiratory failure and ventilatory support was higher, the mortality was lower (11 vs. 21%). Our cohort was older as compared to the total number of SARS-CoV-2 infected patients in Portugal (median age 17 years higher); this was already expected, considering that older patients are more likely to have severe disease, thus requiring hospitalization (35).

Our study focused on IL-6 due to its reported unique role in the cytokine storm occurring in patients with COVID-19, its good correlation to disease severity, the risk of needing mechanical ventilation, or death, and most importantly, because it can be used as a pharmacological target (14, 17, 25, 28–31, 36, 37). Our results demonstrate that increasing the levels of IL-6 correlate to disease severity and identifying particularly well those patients who evolved to more severe stages of COVID-19, a pattern not observed with other markers such as CRP. Indeed, IL-6 seems to be a potential prognostic marker, as we observed several patients in IIb stage with very high IL-6 levels just before entering stage III, 1 or 2 days later, patterns not observed in CRP levels, despite the positive correlation between IL-6 and CRP (27). This differentiation is critical for patients' monitoring, management of resources, or to support important clinical decisions, like discharging patients safely. Some previous studies have already explored the predictive value of IL-6 on several clinical aspects of COVID-19. At least two of them showed that the level of IL-6 at admission is useful to predict the risk of patients needing mechanical ventilation or high-flow oxygen during hospitalization (26, 29). On the other hand, a Spanish group presented a mortality risk model derived from 443 patients, based on IL-6 at admission (the variable with the highest specificity), SpO_2_/FiO_2_ ratio, neutrophil-to-lymphocyte ratio, Lactate dehydrogenase (LDH) level, and age (28). Also, an Italian study suggested a score composed of IL-6 and other six variables as a useful predictor of a composite endpoint of severe COVID-19 and/or in-hospital death (31). Other studies explored the association between IL-6 and the development of lung injury evaluated by CT scan (26, 27). One of these studies evaluated the IL-6 levels throughout hospitalization, recognizing, as in here, the dynamic changes of disease (27). Also, following this concept of variation through time, an Italian retrospective study showed the value of IL-6 combined with CRP and SpO_2_/FiO_2_ in signaling patients that would have clinical deterioration at a very short term (in the first 3 days after admission) (32). Interestingly, the score had also a good performance at predicting death at later timepoints. To the best of our knowledge, our work has followed, for the first time, patients over time and showed that IL-6 has special predictive value in patients hospitalized under oxygen therapy (IIb), identifying those who will worsen and eventually die.

From a clinical point of view, IL-6 levels seem to correlate with respiratory failure (PaO_2_ and SpO_2_), which is in line with recent studies, showing that SARS-CoV-2 activates innate and adaptive immune responses, resulting in the release of IL-6 and other cytokines, increased vascular permeability, and respiratory failure (38). The fact that injured lungs are the major source of IL-6 may explain the correlations observed between the cytokine levels and oxygen needs (38).

The three profiles of patients characterized in our study are not influenced by gender, comorbidities, or treatment. Once the large majority of our patients were treated with drugs that are now known to be ineffective in COVID-19, we believe this IL-6 kinetics reflects the pathophysiology of disease accurately. It will be interesting, in the future, to evaluate the impact of widely used drugs such as remdesivir and corticosteroids on IL-6 kinetics and their correlation with patient survival. In our study, regarding the non-survivors' profile, the continuously increased levels of IL-6 show that these patients are unable to damp inflammation, leading to patient death. Genetic host factors, impaired viral clearance, low levels of type I interferons, increased neutrophil extracellular traps, T-cell exhaustion, and other miscellaneous factors, have been postulated to increase the individual risk of developing a cytokine storm in response to SARS-CoV-2, a phenomenon where IL-6 has a pivotal role, as previously described (38–40). We did find high levels of IL-6 in survivors, usually preceding patients' clinical worsening, but, in all those cases, IL-6 levels rapidly decreased. Concerning the IL-6 peak, it seems to have a short duration. The peak of IL-6 is observed in both survivors' groups, around 7th and 10th days after the onset of symptoms, respectively. Survivors' group 2 also have a peak at 4th day of hospitalization. As survivors' group 1 were admitted 3 days later than survivors' group 2, we may hypothesize that if they came to the hospital 3 days earlier, we would also observe a peak in that group. Although these data are presented in medians, the critical inflammation point of the disease seems to occur around 1 week to 10 days after the onset of symptoms, reinforcing our clinical observations. Other studies found important clinical deterioration and relevant immunologic or pathophysiologic processes occurring during that period: a peak of viral loads in the sputum and the emergence of dual, antibody, and T-cell dependent, immune response (5, 14, 18, 41). We consider that the novel information on the IL-6 rising kinetics reported here is critical to the successful monitoring of hospitalized patients, but also to patients who remain at home and perhaps need more medical attention on that phase. Furthermore, after 10th day of hospitalization, IL-6 levels tend to become close to normal, even in patients that evolved to stage III. Moreover, we hypothesize that there is a narrow period of time in which immunomodulatory drugs may be particularly effective.

Several studies have already been published about the effect of anti-IL6 agents in COVID-19. Most of them result from observational studies, when anti-IL-6 agents were used empirically, according to hospital protocols that were very diverse in terms of severity criteria and timing of administration. So far, only five RCTs using tocilizumab have their results available. An interventional tocilizumab clinical trial (CORIMUNO-TOCI) was developed with 131 patients with moderate, severe, or critical pneumonia, requiring at least 3 L/min of oxygen but without the need for mechanical ventilation (42). RCT-TCZ-COVID-19 was conducted on a sample of 126 patients with pneumonia, PaO_2_/FiO_2_ between 200 and 300 mmHg and an inflammatory phenotype defined by fever and elevated CRP and also excluded patients requiring mechanical ventilation (43). BACC Bay Tocilizumab Trial recruited 242 adult patients with documented SARS-CoV-2 infection with at least two out of three severity criteria (fever, pulmonary infiltrates, or need of supplemental oxygen) and elevation of at least one laboratory parameter associated with inflammation (CRP, ferritin, d-dimer, or lactate dehydrogenase) (44). Patients were excluded if they were receiving more than 10 L of oxygen per minute. COVACTA and EMPACTA enrolled 438 and 391 individuals, respectively (45, 46). In these studies, patients were included if they had evidence of pneumonia and need of supplemental oxygen, with the latter excluding patients needing mechanical ventilatory support.

It seems obvious from this description that IL-6 level was not an inclusion criterion in any of these RCTs. As shown in our study, despite there was some correlation with SpO_2_, a ratio of PaO_2_/FiO_2_, or with CRP, the IL-6 level varies significantly during the infection and has independent meaning, which leaves space to optimizing patient selection. We may hypothesize that therapeutics guided by the IL-6 level, in which randomization would occur only in patients with levels of IL-6 above a certain cut-off, could have produced different, perhaps, better results. In this study, the IL-6 cut-off value as the prognostic value for worse outcome was defined as 86.95 pg/ml, which is in accordance with previous studies. Based on our observations, it is our conviction that IL-6 shall be monitored throughout the infection and not only at admission and that anti-IL-6 therapy should be performed in patients with high IL-6 levels but before the expected peak, to try to avoid clinical deterioration. Even so, pooled data from these RCTs indicate there is a significant reduction in the need for mechanical ventilation when tocilizumab is used, but there is no mortality reduction (33). If we compare to dexamethasone data from RECOVERY, there is a clear difference in sample size, but also in patients' profile: ventilated patients, in whom corticosteroids proved to be more beneficial, were almost always excluded from tocilizumab trials (20). An observational study developed exclusively with critical-ill patients, which compared the evolution of patients at baseline and 1 week after tocilizumab or standard-of-care, showed a significant improvement in FiO_2_, PaO_2_:FiO_2_ and SpO_2_:FiO_2_, total radiographic score, and total vascular score, despite the small sample size (47). We, therefore, hope to have contributed to the development of new studies, where IL-6 levels are considered and used to guide therapy at the individual level.

Finally, we assessed the potential of IL-6 to predict the outcome of patients. First, IL-6 levels were significantly lower in each group of survivors than in non-survivors, while CRP was not significantly different across groups. Then, besides our small sample size, we built up a model with only three variables to predict non-survivors, in which IL-6 was a more significant predictor than CRP or age. Overall, our study demonstrates that, in association with clinical observations, the kinetic measurement of IL-6 during SARS-CoV-2 infection is a crucial tool to predict the prognosis, response to therapy, and outcome of patients with COVID-19.

## Data Availability Statement

The raw data supporting the conclusions of this article will be made available by the authors, without undue reservation.

## Ethics Statement

The studies involving human participants were reviewed and approved by Clinical Board and Ethics Committee. The patients/participants provided their written informed consent to participate in this study.

## Author Contributions

AS, CC, JP, ACas, and RS designed the experiments. AS, AM-F, AO, LD, AM, and ACar performed the experiments. AS, AM-F, and RS analyzed the data. AS, AM-F, CC, JP, ACas, and RS interpreted the results. AS, AM-F, and RS drafted the manuscript and prepared the tables and figures. AS, AM-F, AO, LD, AM, ACar, CC, JP, ACas, and RS revised the paper and approved the final version of the manuscript. All authors contributed to the article and approved the submitted version.

## Conflict of Interest

The authors declare that the research was conducted in the absence of any commercial or financial relationships that could be construed as a potential conflict of interest.

## Abbreviations

IL-6: interleukin-6
CRP: C-reactive protein
COVID-19: coronavirus disease 2019
ROC: receiver operating characteristic.
